# The Effects of Antioxidants and Packaging Methods on Inhibiting Lipid Oxidation in Deep Fried Crickets (*Gryllus bimaculatus*) during Storage

**DOI:** 10.3390/foods11030326

**Published:** 2022-01-24

**Authors:** Jin Gan, Min Zhao, Zhao He, Long Sun, Xian Li, Ying Feng

**Affiliations:** Key Laboratory of Breeding and Utilization of Resource Insects of National Forestry and Grassland Administration, Research Institute of Resource Insects, Chinese Academy of Forestry, Kunming 650233, China; ganjin638@126.com (J.G.); mzhao@caf.ac.cn (M.Z.); zhe999@hotmail.com (Z.H.); sunlong1818@126.com (L.S.); leexian99@163.com (X.L.)

**Keywords:** edible insects, *Gryllus bimaculatus*, processing and preservation, fatty acids, lipid oxidation

## Abstract

This study aimed to investigate the effect of processing methods on inhibiting lipid oxidation of deep fried crickets (*Gryllus bimaculatus*) during storage. Four antioxidants and two packaging methods were used. The effects of different antioxidants and packaging methods on composition of fatty acids, contents of free fatty acids (FFA), peroxide value (PV), and thiobarbituric acid reactive substances (TBARSs) value of deep fried *Gryllus bimaculatus* were analyzed during 150 days of storage. The composition of fatty acids changed and the content of FFA, PV, and TBARs value also increased with the extension of storage time, indicating that the lipid oxidation dominated by oxidation of unsaturated fatty acids could occur in deep fried *Gryllus bimaculatus* during storage. In the same storage period, the total content of FFA, PV, and TBARs value of samples treated with antioxidants and vacuum-filling nitrogen packaging were lower than those of controls, suggesting that antioxidants and vacuum-filling nitrogen packaging have noticeable effects on inhibiting lipid oxidation and improving the quality of deep fried crickets, and dibutyl hydroxyl toluene (BHT) was found as the most effective antioxidant in this study. The results may provide a reliable reference for processing of deep fried edible insects.

## 1. Introduction

Insects are a biological group with the largest number of species, huge biomass, fast reproduction capability, and high food conversion rate on earth. Insects are highly nutritious, containing a large amount of high-quality proteins, high levels of unsaturated fatty acids, and high levels of essential trace elements, such as iron and zinc [1]. Although the data of nutritional composition vary from species to species, most species of insects contain proteins, fat, vitamins, and minerals, corresponding to a human’s nutritional needs [2]. In view of the increase of global population and the decrease of arable land, Meyer-Rochow [3] advocated the use of insects as human food and recommended that the FAO (Food and Agriculture Organization of the United Nations) regarded insects as a potential sustainable food source to deal with global food security problems, and encouraged more use of insects in daily diets [4]. More than 2000 kinds of insects are eaten in more than 110 countries/regions worldwide. The commonly edible insects include Coleoptera, Lepidoptera, Hymenoptera, Orthoptera, and Hemiptera. Among Orthoptera insects, crickets are the most widely consumed insect species [5].

Crickets belong to Gryllidae of Grylloidea in Orthopera, which are widely distributed universally. The commonly consumed varieties are *Brachytrupes membranaceus*, *Gryllus similis*, *Gryllus bimaculatus*, *Gryllotalpa orientalis*, and *Acheta domesticus* [6]. Crickets are also rich in nutrients, with a protein content of 55–70% [7], and the proportion of unsaturated fatty acids (UFAs) is more than 60% in lipids, which may even reach 80% in some varieties, such as *Gryllus testaceus* Walker and *Teleogryllus emma* [8,9]. Regarding minerals, calcium, iron, zinc, and copper contents in crickets are higher than those of conventional foods of animal origin, but not dangerously so [9]. Moreover, the low chitin content and hardness of crickets grown for six to seven weeks may lead to good taste and palatability [6]. Therefore, crickets promote the development of breeding industry in some countries in Asia, Europe, America, Australia, and Africa continents, especially in tropical regions (e.g., Southeast Asia). As reported previously, the total annual output of feeding crickets in Thailand is within 3000–7000 tons [10], and although the cricket farming scale in Cambodia and Laos is smaller than that in Thailand, it is promptly expanding and developing [7]. In Korea, cricket farming has a history of about 20 years. Farmed crickets are mainly used as feed for livestock and aquaculture, and the number of farmed crickets will also increase due to the use of cricket flour as a protein-rich additive to flour in the baking industry [11]. In recent years, cricket farming as the basis of food processing has markedly attracted scholars’ attention as a result of the recognition of the nutritional value and food safety of edible crickets. Cambodian Center for Livestock and Agriculture Development evaluated the influences of different types of feed on the survival rate and growth of crickets [12]; Kenya aimed to introduce cool and high-altitude areas to expand the production of crickets [13]; United States Department of Agriculture—Agricultural Research Service National Biological Control Laboratory proposed an optimal harvesting age for reducing costs of cricket production by determining food conversion efficiency at different ages [14].

The development of cricket farming provides the possibility for exploiting and industrialization of cricket-based foods. Crickets are also consumed in making bread, biscuits, noodles, and meat sauce as additives [15,16,17,18]. However, deep frying is a common method of processing of crickets [19], and bagged fried cricket is a popular snack food in Thailand, Laos, and other countries. China is one of the first countries known to consume insects as food [20]. Although crickets have not been approved as a new resource of food in China, cricket farms and companies have recently appeared in some regions of China, and the scale is expanding. The breeding varieties are mainly *Gryllus bimaculatus* and *Acheta domesticus*, in which *Gryllus bimaculatus* is more popular due to its short life-cycle, being stronger, and having a cold resistance. In addition to being used as pet feed and serving at restaurants, farmed crickets are mainly processed with deep fried and packaging in small workshops, where they then attract consumers’ attention because of their noticeable nutritional level and delicious taste. However, the lipid oxidation occurs easily during processing and storage as a result of abundance of UFAs in edible insects, influencing the quality and flavor of products in the period of storage. This is a prominent and urgent problem that needs to be eliminated in the production of edible insect, especially deep fried edible insects.

Therefore, regarding *Gryllus bimaculatus* as a food source, four antioxidants and two packaging methods were used in the present study, and fatty acid profile, free fatty acid content, and lipid oxidation indices, such as peroxide value (PV) and thiobarbituric acid reactive substances (TBARs), of deep fried crickets were analyzed during 150 days of storage. The effects of antioxidants and packaging methods on lipid oxidation were studied for improving processing technology of deep fried crickets.

## 2. Materials and Methods

### 2.1. Chemicals and Materials

A standard mixture of 37 fatty acid methyl esters, glyceryl triundecanoate standard, and undecanoic acid standard were purchased from Sigma-Aldrich Co., Ltd. (Shanghai branch, China); petroleum ether, n-heptane, boron trifluoride methanol solution, chloroform, and 2-isopropanol were purchased from Kunming Beijie Technology Co., Ltd. (Kunming, China); four antioxidants, including dibutyl hydroxyl toluene (BHT), rosemary extract (ET), and tert-butylhydroquinone (TBHQ), were purchased from Zhejiang Yinuo Biotechnology Co., Ltd. (Hangzhou, China), and *Phyllanthus emblica* polyphenol (PEP) was prepared by our laboratory; The packing bags made of PET/PE (polyethylene terephthalate/polyethylene, 0.16 mm of total thickness) and having excellent air tightness, oil resistance and fragrance retention, were purchased from Cangzhou Jingtian Plastic Industry Co., Ltd. (Cangzhou, China)

### 2.2. Preparation of Cricket Samples

The fresh *Gryllus bimaculatus* used in the experiment were provided by Yunnan Kuoyang Agricultural Science and Technology Co., Ltd. (Kunming, China), and identified by Research Institute of Resource Insects, Chinese Academy of Forestry (Kunming, China). The feeding life-cycle of crickets was 6–7 weeks. After fasting for 2 days, they were frozen at −18 °C for 1 h. The fresh *Gryllus bimaculatus* contain 71% water, 16.99% protein, and 9.98% lipid.

### 2.3. Preparation of Deep Fried Crickets

The dead crickets were washed with water and divided into 6 portions. One portion was 2 kg and that was deeply fried at 160 °C for 5 min in 20 kg of palm oil containing different antioxidants. The addition proportion of the four antioxidants was determined according to the maximum use limit of each antioxidant in the oil, and the amount of antioxidants used was based on the weight of palm oil. The lipid contents before and after deep frying of the crickets were 34.43% and 51.55% of dry weight, respectively. Two packaging methods were used for the deep fried crickets, including non-vacuum sealed packaging and vacuum-filling nitrogen packaging. Non vacuum packaging samples in bags made of PET/PE (polyethylene terephthalate/polyethylene, 0.16 mm of total thickness) were air-packed (50 g sample per bag) using a sealing machine (Guangzhou Feipu Packaging Machinery Co., Ltd., Guangzhou, China). Vacuum-filling nitrogen packaging samples in bags made of PET/PE were packed (50 g sample per bag) by vacuumizing and filling in with nitrogen using a vacuum sealing machine equipped a nitrogen cylinder (Zhejiang Baochun Packaging Machinery Co., Ltd., Wenzhou, China). The treatment methods of 6 samples were as follows: BHTV—0.2% BHT + vacuum-filling nitrogen packaging; ETV—0.5% ET + vacuum-filling nitrogen packaging; PEPV—0.5% PEP + vacuum-filling nitrogen packaging; TBHQV—0.2% TBHQ + vacuum-filling nitrogen packaging; control 1—no antioxidant + vacuum-filling nitrogen packaging; and control 2—no antioxidant + non vacuum sealed packaging. The samples were stored at room temperature in a dark place for the analysis of indices every 30 days.

### 2.4. Lipid Extraction

The lipids were extracted using Soxhlet extractor, in which 10 g of crushed sample and 300 mL of petroleum ether were transferred into a 500 mL flat bottom flask, and maintained in a 40 °C water bath for 8 h. The extract solution was concentrated using a rotary evaporator (Eyela, Tokyo, Japan) to obtain the lipid extract.

### 2.5. Analysis of Composition of Fatty Acids

As described by Chinese national standard GB 5009.168-2016 [21], briefly, glyceryl triundecanoate was used as an internal standard, and 60 mg of lipid extract and 2 mL of glyceryl triundecanoate solution (2.5 mg/mL) were mixed with 8 mL of 2% sodium hydroxide methanol solution. The mixture was incubated for 2 h on a water bath at 80 °C for saponification, and then 7 mL of 15% boron trifluoride methanol solution was added into the mixture and continually incubated for 6 min to achieve methyl esterification. After cooling, the esterification solution was mixed and shaken with 30 mL n-heptane and 30 mL saturated sodium chloride solution, kept for layering, and the upper solution was taken out for analysis.

Fatty acid methyl esters were analyzed by gas chromatography equipped with a polydicyanopropyl siloxane strong polar stationary phase capillary column (100 m × 250 μm ID × 0. 2 μm film). The temperature of the injection port and the detector was set to 270 °C and 280 °C, respectively. The carrier gas was nitrogen, and the flow rate was 1.5 mL/min. The injection volume was 1 mL, and the split ratio was 10:1. The procedure was completed at the following thermal conditions: The initial temperature of 100 °C was kept for 13 min, and it increased to 180 °C at 10 °C/min and maintained for 6 min, and then the temperature was elevated to 200 °C at 1 °C/min and maintained for 20 min. Identification of fatty acid methyl esters was performed by comparing the retention time of the standard mixture of fatty acid methyl esters. The fatty acids were quantified by an internal standard, and the proportion of each fatty acid was calculated as the ratio of each fatty acid content to the total fatty acid content, and the result was expressed as percentage of total fatty acids. Each sample was analyzed in triplicate.

### 2.6. Determination of the Contents of Free Fatty Acids (FFAs)

Free fatty acids were separated by Wang et al.’s method [22], in which 240 mg of lipid extract was dissolved in 12 mL of chloroform, and 10 mL of chloroform solution was loaded onto an aminopropyl-silica minicolumn (500 mg/3 mL), which was previously activated with 10 mL of chloroform. The minicolumn was eluted with 10 mL of chloroform/2-isopropanol (2/1, *v*/*v*), and the eluent was discarded. The free fatty acids were eluted by 15 mL of ether solution, containing 2% acetic acid *(w*/*w*), the solvent was removed from eluent using a rotary evaporator, and the residue was free fatty acid.

The free fatty acids and 2 mL of undecanoic acid solution (50 μg/mL), as an internal standard, were mixed with 7 mL of 15% boron trifluoride methanol solution, and the mixture was incubated for 6 min on a water bath at 80 °C for methyl esterification. The cooled esterification solution was mixed and shaken with 20 mL n-heptane and 20 mL saturated sodium chloride solution, kept for layering, and the upper solution was taken out for analysis. Fatty acid methyl ester was analyzed according to the above-mentioned methods, and the result was expressed as mg/100 g sample. Each sample was analyzed in triplicate.

### 2.7. Determination of Peroxide Value (PV)

PV was determined according to Chinese national standard GB 5009.227-2016 [23]. Specifically, 10 g of crushed sample was mixed with 20 mL petroleum ether and was kept for 12 h. The filtrate was evaporated to remove petroleum ether under vacuum using a rotary evaporator in a water bath at 35 °C. The residue was dissolved in 30 mL of chloroform-glacial acetic acid mixture (2/3, *v*/*v*) and transferred to a 250 mL iodine-measuring bottle, and 1 mL of saturated potassium iodide solution was then added. After completion of reaction for 3 min in a dark place, 100 mL of deionized water and 1 mL of 1% starch solution, as an indicator, were added, and the reaction solution was titrated with sodium thiosulfate solution immediately until the intense blue color disappeared and solution became canary yellow. The blank test was performed according to the above-mentioned method. Each sample was analyzed in triplicate. The PV was calculated by Equation (1).

(1) $$X=\frac{\left(V-V_{0}\right)\times c\times 0.1269\times 1000}{m}\times 100$$

where X represents peroxide value (mg/100 g); *V* and *V*_0_ are the volume of sodium thiosulfate solution consumed by the sample and the blank, respectively (mL); c is the concentration of sodium thiosulfate solution (mol/L); 0.1269 denotes the mass of iodine equivalent to 1 mL of 1 mol/L sodium thiosulfate solution; and *m* indicates the sample weight (g).

### 2.8. Determination of Thiobarbituric Acid Reactive Substances (TBARs)

TBARs assay was performed according to Chinese national standard GB 5009.181-2016 [24]. First, 1,1,3,3-tetraethoxypropane was used as an internal standard. Then, 5 g of crushed sample and 50 mL of 7.5% trichloroacetic acid were placed into a 150 mL of conical flask, and shaken at 150 rpm for 30 min in an Ecotron shaking incubator (INFORS, Basel, Switzerland). The mixture was filtered with paper filters. Next, 5 mL of filtrate and standard solution (0.01, 0.05, 0.1, 0.15, 0.25 μg/L) was mixed with 5 mL of 0.02 mol/L thiobarbituric acid solution, respectively, and 5 mL of deionized water was used as blank. The mixture was incubated in a water bath at 90 °C for 30 min. The absorbance of mixture was measured using a microplate reader (Thermo Fisher Scientific, Waltham, MA, USA) at 532 nm after cooling. The standard curve was established with the standard solution concentration as the horizontal coordinate and the absorbance as the vertical coordinate. The TBARs value of the sample was calculated according to the standard curve and the dilution ratio of the sample. The result was expressed as milligrams malondialdehyde per kilogram of sample (mg MDA/kg). Each sample was analyzed in triplicate.

### 2.9. Statistical Analysis

All experiments were performed in triplicate. All data are expressed as mean ± standard deviations (SD). One-way analysis of variance (ANOVA) was used for comparing differences using the SPSS 13.0 software (IBM, Armonk, NY, USA). Bonferroni correction and Duncan’s test were used for multiple comparisons.

## 3. Results

### 3.1. Fatty Acid Compositions of Raw Gryllus bimaculatus and Palm Oil

In order to analyze the changes of fatty acids in the samples after deep frying, the fatty acid compositions of raw *Gryllus bimaculatus* and palm oil were determined. The main fatty acids of different species of crickets are palmitic acid (C16:0), oleic acid (C18:1) and linoleic acid (C18:2), and the content of oleic acid in *Gryllus bimaculatus* is generally higher than that of other varieties [25]. As shown in Table 1, the fatty acid of *Gryllus bimaculatus* includes myristic acid (C14:0), palmitic acid (C16:0), palmitoleic acid (C16:1), stearic acid (C18:0), oleic acid (C18:1), linoleic acid (C18:2), and linolenic acid (C18:3). Among them, palmitic acid, oleic acid and linoleic acid are the main fatty acids of *Gryllus bimaculatus*. The results also showed that the proportion of UFAs was 65.79%, mainly consisting of oleic acid (22.75%) and linoleic acid (41.75%), the proportion of saturated fatty acids (SFAs) was 34.21%. Compared with the previously reported results on the analysis of fatty acids in *Gryllus bimaculatus* [26,27], the composition and content of main fatty acids were consistent, except for a trace of arachidic acid (C20:0) and arachidonic acid (C20:1), which were not detected in the present study.

Palm oil contains five fatty acids—myristic acid, palmitic acid, stearic acid, oleic acid, and linoleic acid (Table 1)—and the main fatty acids are palmitic acid, oleic acid, and linoleic acid, which are the same as *Gryllus bimaculatus*. However, the contents of palmitic acid and oleic acid are remarkably higher than those of *Gryllus bimaculatus*, and the content of linoleic acid is much lower than that of *Gryllus bimaculatus*.

### 3.2. Changes in Fatty Acid Composition of Deep Fried Samples

The fatty acid composition of lipids is a critical indicator of nutritional value, and it is associated with the oxidative stability of lipids. The fatty acids of deep fried samples with different treatments during storage are summarized in Table 2. The results indicated that the fatty acid composition of deep fried crickets (0 days) significantly varied compared with the raw materials (Table 1). Palmitoleic acid and linolenic acid were not found, and the contents of palmitic acid and oleic acid increased significantly, while the content of linoleic acid markedly decreased, and the content of stearic acid was reduced as well. The proportion of UFAs decreased to 55% (Table 2) from 65.79% in raw samples (Table 1). Deep fried samples contain a large amount of palm oil after frying due to the penetration of palm oil into samples during frying. Therefore, the compositions and contents of fatty acids were similar to those of palm oil.

As shown in Table 2, during the storage period (150 days), there were significant differences between the contents of fatty acids in the same sample at different periods of storage, including palmitic acid, stearic acid, oleic acid, linoleic acid, ΣUFAs, ΣSFAs, and UFAs/SFAs in all samples (*p* < 0.001), as well as myristic acid in PEPV, TBHQV, control 1, and control 2 (*p* < 0.001). Meanwhile, the decline of ΣUFAs, the increase of ΣSFAs, and the decrease of UFAs/SFAs in all samples during storage were also observed, and the changes of ΣUFAs and ΣSFAs were the results of the decline of the content of oleic acid and the raise of the contents of myristic acid and palmitic acid. These results suggested that the oxidation of UFAs in the deep fried *Gryllus bimaculatus* occurred during storage, and UFAs were found more susceptible to oxidation than SFAs.

Regarding the differences in samples with different treatments in the same storage time, Table 2 shows that there were significant differences between the contents of stearic acid, oleic acid, and linoleic acid from different samples in the same storage time (*p* < 0.001). The content of myristic acid in different samples significantly changed after storing for 60 and 150 days (*p* < 0.01 and *p* < 0.05, respectively). The difference in the content of palmitic acid from different samples was statistically significant after storing for 60, 90, and 150 days (*p* < 0.01, *p* < 0.05, and *p* < 0.001, respectively). For ΣUFAs, ΣSFAs, and UFAs/SFAs, there was no significant difference among samples within 30–90 days after storage (*p* > 0.05), while the difference gradually increased with the extension of storage time. After 120 and 150 days of storage, the difference was significant (*p* < 0.01 and *p* < 0.001, respectively). The results indicated that different antioxidants and packaging treatments had certain effects on the changes of compositions of fatty acids during storage, and the effects gradually increased with the extension of storage time.

### 3.3. Contents of FFAs in Deep Fried Samples

FFA is attributed to the oxidation and hydrolysis of lipids under lipase and oxygen. The results from the analysis of FFAs in deep fried *Gryllus bimaculatus* (Table 3) indicated that palmitic acid, stearic acid, oleic acid, and linoleic acid were detected in samples. The contents of these FFAs from the same sample in different periods of storage were significantly different (*p* < 0.05, *p* < 0.01, and *p* < 0.001, respectively). The contents of FFAs noticeably increased with the prolongation of storage time, and the contents of ΣSFFAs were markedly higher than those of ΣUFFAs. The contents of a single FFA and total FFAs in BHTV reached the maximum values after 90 or 120 days of storage, and then decreased slightly. The contents of a single FFA and total FFAs in other samples basically reached the peak after 150 days of storage, due to the continuous accumulation of FFAs with the oxidation and hydrolysis of lipids.

As presented in Table 3, the contents of a single FFA and total FFAs in different samples significantly varied (*p* < 0.05, *p* < 0.01, and *p* < 0.001, respectively) at the same storage time. The ΣUFFAs, ΣSFFAs, and total content of FFAs in control 2 were the highest among all the samples, especially the total content of FFAs (218.45 and 227.53 mg/100 g) was markedly higher than that of other samples after 120 and 150 days of storage. Meanwhile, the total content of FFAs in BHTV, TBHQV, and PEPV was lower than that of control 1 after 150 days of storage. These results demonstrated that antioxidants and vacuum-filling nitrogen packaging had inhibitory effects on the lipid oxidation in deep fried *Gryllus bimaculatus*. The total content of FFAs in BHTV was the lowest among samples treated with antioxidant, suggesting that the effect of BHT on lipid oxidation in deep fried *Gryllus bimaculatus* was more noticeable than that of other antioxidants.

### 3.4. PV of Deep Fried Samples

**Figure 1:**
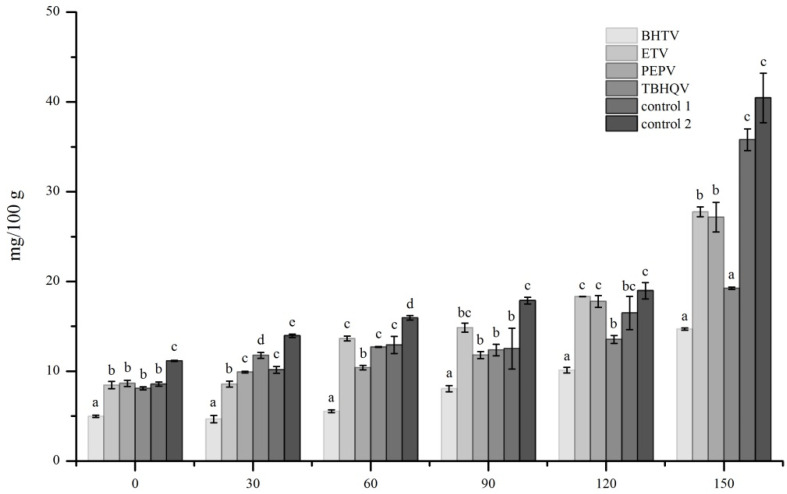
Changes in peroxide values (PVs) of deep fried *Gryllus bimaculatus*. BHTV = 0.2% BHT + vacuum-filling nitrogen packaging; ETV = 0.5% ET + vacuum-filling nitrogen packaging; PEPV = 0.5% PEP + vacuum-filling nitrogen packaging; TBHQV = 0.2% TBHQ + vacuum-filling nitrogen packaging; control 1 = no antioxidants + vacuum-filling nitrogen packaging; control 2 = no antioxidant + non vacuum sealed packaging; different lowercase letters on the column chart showed significant differences in the same storage time (*p* < 0.001).

The PV was used to measure the primary lipid-oxidation products, especially hydroperoxides [28], which could be further decomposed into low-molecular-weight substances, such as aldehydes, ketones, and acids. The level of lipid oxidation can be judged from PV. Figure 1 shows that the PVs of all samples increased during storage, and the PVs of different samples in the same period were significantly different (*p* < 0.001). The PV of control 2 was the highest in each storage time point. The PVs of control 1 and control 2 were markedly higher than those of other samples after 150 days of storage (35.79 and 40.05 mg/100 g, respectively). Among samples that were treated with antioxidants, the PVs of BHTV and TBHQV were lower than those of other samples, which could be corresponded to the results of analysis of FFAs, indicating that the methods of using antioxidants and vacuum-filling nitrogen packaging could inhibit the oxidation of lipids, in which the effects of BHT and TBHQ were more noticeable.

### 3.5. TBARs of Deep Fried Samples

**Figure 2:**
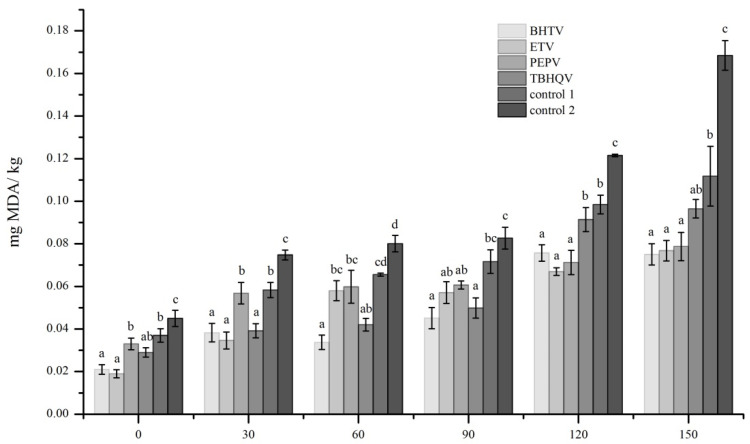
Changes in thiobarbituric acid reactive substances (TBARs) values of deep fried *Gryllus bimaculatus*. BHTV = 0.2% BHT + vacuum-filling nitrogen packaging; ETV = 0.5% ET + vacuum-filling nitrogen packaging; PEPV = 0.5% PEP + vacuum-filling nitrogen packaging; TBHQV = 0.2% TBHQ + vacuum-filling nitrogen packaging; control 1 = no antioxidants + vacuum-filling nitrogen packaging; control 2 = no antioxidant + non vacuum sealed packaging; different lowercase letters on the column chart showed significant differences in the same storage time (*p* < 0.001).

TBARs is an important indicator of oxidation of fatty acids during storage and processing, which could be characterized with the content of malondialdehyde (MDA) [29]. MDA is one of the main end-products of lipid oxidation. A continuous increase in TBARs values of all samples was observed during storage (Figure 2), and the differences in TBARs values of different samples at the same storage time were statistically significant (*p* < 0.001), in which the TBARs values of control 1 and control 2 were higher than those of other samples, especially the differences in TBARs values of control 2 and other samples increased gradually with the extension of storage time. After 150 days of storage, the TBARs value of control 2 was notably higher than that of other samples. In samples treated with antioxidants, the TBARs values of BHTV and TBHQV were low after 90 days of storage, and the TBARs values of BHTV, ETV, and PEPV were approximately lower than those of TBHQV after 150 days of storage. The results indicated that the methods of using antioxidants and vacuum-filling nitrogen packaging could be advantageous for controlling lipid oxidation in deep fried *Gryllus bimaculatus*. Among antioxidants used in the present study, BHT, ET, and PEP were more appropriate for reducing TBARs value.

## 4. Discussion

Cooking and processing methods have a great influence on the composition of fatty acids in the products. In the frying process, the composition of fatty acids in the deep fried products may greatly change compared with the raw materials due to absorbing a large amount of frying oil. Weber et al. [29] and Garcia-Arias et al. [30] analyzed fatty acids of silver catfish and sardine fillets processed by different methods, and found that the composition of fatty acids in samples processed by frying and baking did not significantly vary, while the ratio of UFAs to SFAs in deep fried fish slices significantly increased as a result of the high contents of unsaturated fats in soybean oil used for frying. Palm oil is widely used in the fried food industry and the fried cricket enterprises due to its stability in the frying process, which is not easy to be hydrolyzed and oxidized, and can make the fried products have a good taste, as well as the price of palm oil is relatively cheap. As a result of its stability, palm oil has little impact on the quality of fried food, but the fatty acid composition of the fried food may change in different levels due to the adsorption of products to palm oil. In this study, fried crickets may adsorb about 17% of palm oil, therefor, the data from the analysis of composition of fatty acids in deep fried *Gryllus bimaculatus* were consistent with previously reported results, in which the composition of fatty acids in processed samples significantly differed from that of raw materials, and the contents of palmitic acid and oleic acid increased, while the contents of stearic acid and linoleic acid decreased, as the palm oil used for frying crickets has high contents of palmitic acid and oleic acid and low contents of stearic acid and linoleic acid.

During the processing and storage of animal products, the oxidation and hydrolysis of lipid occur easily under the action of lipase and oxygen. The stability of various fatty acids is different, and polyunsaturated fatty acids are more susceptible to oxidation, followed by monounsaturated fatty acids, while SFAs are the most stable type [31]. In the present study, the decline of ΣUAFs and the increase of ΣSFAs in deep fried cricks during storage were observed, and the analysis of FFAs showed that the contents of ΣSFFAs were higher than those of ΣUFFAs. These results also proved that UFAs in deep fried crickets were unstable and prone to oxidation.

As a result of lipid oxidation, UFAs containing double bonds with very unstable properties is hydrolyzed to hydroperoxides [32]. The peroxides accumulate gradually when the generation of peroxides is greater than their decomposition, resulting in the increase of PV. Therefore, TBARs value also increases gradually. The results of the current study showed that PV and TBARs values of deep fried crickets increased during 150 days of storage, which are consistent with the results of previous studies on beef [33], chicken [34], pork [35], and fish [28], indicating that the oxidation of fatty acids in deep fried crickets is similar to other meat, and is consistent with the variations of fatty acids and FFAs.

Compared with other meat products, such as pork [32], beef [33], and goose [22], edible insects have higher contents of UFAs, leading to lipid oxidation easily. Although there are relatively few studies on fatty acid oxidation of edible insects during processing and storage, a number of relevant researches have been conducted. For instance, Kinyuru [36] studied the composition of fatty acids in deep fried termites and long-horned grasshoppers, and it was revealed that the proportion of SFAs increased, whereas the proportion of UFAs significantly decreased during frying, indicating the occurrence of lipid oxidation and degradation. The temperature of processing has a significant effect on the composition of fatty acids of crickets, and the contents of polyunsaturated fatty acids in Acheta domesticus decreased from 45% to 30%, while the contents of monounsaturated fatty acids increased from 21% to 39% under freeze-drying and heat-drying respectively [37]; for black crickets, the content of palmitic acid in the sample dried at 120 °C was markedly higher than that of freeze-drying, while the contents of linoleic acid and linolenic acid were significantly lower than those of freeze-drying [38]. Kim et al. [39] and Ssepuuya et al. [40] analyzed the effects of storage conditions on lipid oxidation of edible insects, although there was no significant difference in the contents of main fatty acids of *Gryllus bimaculatus* powder after storage at 40 °C for six months, the indicator of lipid oxidation such as acid value varied significantly; vacuum packaging and refrigeration could inhibit the lipid oxidation of fried grasshoppers and prolong the shelf-life to 22 weeks, in which the PV of the product was <21.50 mEq O_2_/kg, and the TBARs was <0.079 mg MDA/kg 40]. These studies demonstrated that different degrees of lipid oxidation and decomposition may occur in edible insects during processing and storage, which are similar to other animal foods. Lipid oxidation is an important factor, influencing the quality and shelf-life of animal foods.

Antioxidants inhibit the oxidation of lipids by scavenging the peroxide reaction matrix, complexing metal ions, reducing the concentration of active oxygen and blocking the dehydrogenation of fatty acids [41,42]. Synthetic antioxidants commonly used in meat processing include dibutyl hydroxyl toluene (BHT), tert-butylhydroquinone (TBHQ), and butyl hydroxyanisole (BHA), which have good antioxidant effects and can delay lipid oxidation by eliminating free radicals and chelating metal ions to increase the shelf life of meat products [43]. Natural antioxidants are increasingly used in meat products due to they are safer than chemically synthesized antioxidants, in which rosemary extract is more widely used [44,45]. The strong antioxidant activity of rosemary is related to substances such as carnosic acid, carnosol, diterpenes and rosemary diphenols [46], which slow down the oxidation process by combining with hydroxyl radicals and peroxides to form stable quinones [47,48] and chelating metal ions [49]. Plant polyphenols have been proven to have a good inhibitory effect on lipid oxidation in meat products [50], and *Phyllanthus emblica* polyphenols have activities such as scavenging free radicals and anti-lipid oxidation [51]. Therefore, two chemically synthesized antioxidants, including BHT and TBHQ, and two natural antioxidants, including oil-soluble rosemary extract (ET) and *Phyllanthus emblica* polyphenols (PEP), were used for the investigation in this study. The analysis results of free fatty acids content and PV value show that the antioxidant effects of BHT and TBHQ are better than that of ET and PEP, while the antioxidant effects of BHT, ET, and PEP are better than that of TBHQ in the analysis results of TBARs, in which the most effective antioxidant is BHT. Although some studies have reported that the antioxidant effects of some natural antioxidants in meat products are equivalent to BHT [52], and even better than BHT [53], the antioxidant effects of chemically synthesized antioxidants are significantly better than that of natural antioxidants in this study. The difference between the results of this study and the literatures may be related to different processing methods. The processing method in this study is high-temperature frying. At high temperatures, natural antioxidants are more unstable and easier to decompose. Therefore, chemical synthesis of antioxidants has greater advantages in the processing of fried food. In this study, only single antioxidants were carried out, and the combination of different antioxidants may have better effects, which can be further studied.

## 5. Conclusions

In order to study and inhibit lipid oxidation in deep fried *Gryllus bimaculatus*, four antioxidants were added to the palm oil used for deep frying, and the non-vacuum sealed packaging and vacuum-filling nitrogen packaging methods were used. Besides, the composition of fatty acids, contents of FFAs, PV, and TBARs value of samples that were treated with different methods during 150 days of storage were analyzed. The results showed that the contents of UFAs decreased, while the contents of SFAs increased. Meanwhile, the total content of FFAs, PV, and TBARs value increased during 150 days of storage, indicating that the lipid oxidation dominated by oxidation of UFAs could occur in deep fried *Gryllus bimaculatus* during storage. Additionally, at the same storage time, there were significant differences in the contents of UFAs and SFAs, as well as the total content of FFAs, PV, and TBARs value of samples treated with different methods. The total content of FFAs, PV, and TBARs value of samples treated with antioxidants and vacuum-filling nitrogen packaging were lower than those of the control, suggesting that antioxidants and vacuum-filling nitrogen packaging could significantly inhibit the lipid oxidation in deep fried *Gryllus bimaculatus*, which is an effective method to improve the quality and shelf-life of deep fried crickets, and BHT was found as the most effective antioxidant in the present study.

## Figures and Tables

**Table 1:** Composition of fatty acids in fresh *Gryllus bimaculatus* and palm oil (% of total fatty acids) ^1^.

	Content of Fatty Acids (% of Total Fatty Acids)	
	*Gryllus bimaculatus*	Palm Oil
Myristic acid (C14:0)	0.58 ± 0.01	0.97 ± 0.02
Palmitic acid (C16:0)	24.31 ± 0.14	40.67 ± 0.2
Palmitoleic acid (C16:1)	0.44 ± 0.00	-
Stearic acid (C18:0)	9.32 ± 0.07	4.24 ± 0.03
Oleic acid (C18:1)	22.75 ± 0.09	42.8 ± 0.04
Linoleic acid (C18:2)	41.75 ± 0.23	11.23 ± 0.03
Linolenic acid (C18:3)	0.85 ± 0.01	-
ΣUFA ^2^	65.79 ± 0.19	54.03 ± 0.07
ΣSFA ^3^	34.21 ± 0.2	45.89 ± 0.21
UFA/SFA	1.92 ± 0.02	1.18 ± 0.01

^1^ “-” means that this fatty acid was not detected; ^2^ “UFA” indicates unsaturated fatty acids; ^3^ “SFA” indicates saturated fatty acids.

**Table 2:** Composition of fatty acids in deep fried *Gryllus bimaculatus* during storage (% of total fatty acids) ^1^.

Fatty Acids	Treatments ^2^	Storage Time/Days						Sign. ^3^
		0	30	60	90	120	150	
Myristic acid (C14:0)	BHTV	0.88 ± 0.00	1.01 ± 0.02	0.73 ± 0.01 ^B^	0.85 ± 0.00	1.34 ± 0.53	0.93 ± 0.02 ^AB^	n.s.
	ETV	0.89 ± 0.00	1.02 ± 0.28	0.76 ± 0.00 ^B^	0.86 ± 0.02	1.04 ± 0.05	0.94 ± 0.00 ^AB^	n.s.
	PEPV	0.87 ± 0.00 ^b^	0.93 ± 0.03 ^c^	0.80 ± 0.01 ^Ba^	0.86 ± 0.01 ^b^	0.96 ± 0.02 ^c^	0.94 ± 0.01 ^ABc^	***
	TBHQV	0.89 ± 0.03 ^b^	0.90 ± 0.02 ^bc^	0.81 ± 0.01 ^Ba^	0.89 ± 0.01 ^b^	0.93 ± 0.01 ^c^	0.93 ± 0.01 ^ABc^	***
	control 1	0.87 ± 0.00 ^b^	0.94 ± 0.06 ^b^	0.68 ± 0.07 ^Aa^	0.85 ± 0.01 ^b^	0.95 ± 0.06 ^b^	0.95 ± 0.01 ^Ab^	***
	control 2	0.87 ± 0.00 ^b^	090 ± 0.02 ^c^	0.81 ± 0.01 ^Ba^	0.87 ± 0.01 ^b^	0.92 ± 0.02 ^c^	0.91 ± 0.01 ^Bc^	***
	Sign.	n.s.	n.s.	**	n.s.	n.s.	*	
Palmitic acid (C16:0)	BHTV	38.4 ± 0.04 ^b^	37.69 ± 0.06 ^a^	39.90 ± 0.10 ^Cd^	39.26 ± 0.17 ^ABc^	40.55 ± 0.37 ^e^	40.66 ± 0.21 ^BCe^	***
	ETV	38.37 ± 0.02 ^b^	37.84 ± 0.08 ^a^	39.32 ± 0.11 ^BCd^	39.07 ± 0.20 ^ABc^	40.16 ± 0.24 ^e^	40.68 ± 0.02 ^BCf^	***
	PEPV	37.84 ± 0.09 ^a^	37.70 ± 0.26 ^a^	39.09 ± 0.15 ^ABCc^	38.57 ± 0.21 ^Ab^	39.73 ± 0.10 ^d^	40.75 ± 0.06 ^BCe^	***
	TBHQV	37.94 ± 0.06 ^ab^	37.80 ± 0.06 ^a^	38.49 ± 0.26 ^Ab^	39.39 ± 0.40 ^ABc^	39.67 ± 0.47 ^c^	40.38 ± 0.38 ^Bd^	***
	control 1	38.3 ± 0.47 ^b^	37.41 ± 0.07 ^a^	39.51 ± 0.72 ^BCc^	39.56 ± 0.19 ^Bc^	40.45 ± 0.03 ^d^	41.01 ± 0.06 ^Cd^	***
	control 2	38.00 ± 0.01 ^a^	37.77 ± 0.29 ^a^	39.00 ± 0.22 ^ABb^	39.04 ± 0.41 ^ABb^	39.77 ± 0.45 ^c^	39.87 ± 0.08 ^Ac^	***
	Sign.	n.s.	n.s.	**	*	n.s.	***	
Stearic acid (C18:0)	BHTV	5.37 ± 0.00 ^Dd^	5.17 ± 0.01 ^Ac^	4.43 ± 0.06 ^BCa^	5.07 ± 0.02 ^CDb^	5.15 ± 0.01 ^Bc^	5.25 ± 0.02 ^Cd^	***
	ETV	5.21 ± 0.01 ^Bc^	5.45 ± 0.02 ^De^	4.29 ± 0.07 ^Ba^	4.98 ± 0.01 ^BCb^	5.05 ± 0.02 ^Ab^	5.01 ± 0.02 ^ABb^	***
	PEPV	5.43 ± 0.01 ^Ee^	5.28 ± 0.05 ^Bd^	4.76 ± 0.01 ^Da^	5.17 ± 0.04 ^Dec^	5.19 ± 0.02 ^Bc^	5.08 ± 0.04 ^Bb^	***
	TBHQV	5.19 ± 0.01 ^Ad^	5.16 ± 0.02 ^Ad^	4.62 ± 0.05 ^CDa^	4.91 ± 0.03 ^Bb^	5.06 ± 0.06 ^Ac^	5.00 ± 0.07 ^ABc^	***
	control 1	5.29 ± 0.02 ^Cc^	5.16 ± 0.02 ^A^	3.92 ± 0.29 ^Aa^	4.70 ± 0.16 ^Ab^	5.22 ± 0.06 ^Bc^	4.98 ± 0.02 ^Ac^	***
	control 2	5.44 ± 0.01 ^Ed^	5.36 ± 0.04 ^Cc^	5.18 ± 0.03 ^Ea^	5.27 ± 0.04 ^Eb^	5.20 ± 0.02 ^Ba^	5.28 ± 0.01 ^Cb^	***
	Sign.	***	***	***	***	***	***	
Oleic acid (C18:1)	BHTV	40.47 ± 0.02 ^Ac^	41.40 ± 0.13 ^BCd^	40.45 ± 0.03 ^Bc^	40.09 ± 0.19 ^Ab^	37.45 ± 0.09 ^Aa^	37.39 ± 0.16 ^Aa^	***
	ETV	41.07 ± 0.03 ^Bd^	40.46 ± 0.41 ^Ac^	41.66 ± 0.06 ^Ce^	40.55 ± 0.22 ^Bc^	38.25 ± 0.08 ^Bb^	37.84 ± 0.03 ^Ba^	***
	PEPV	40.36 ± 0.06 ^Abc^	40.97 ± 0.37 ^Abd^	40.47 ± 0.10 ^Bc^	40.00 ± 0.22 ^Ab^	37.88 ± 0.23 ^Aa^	37.82 ± 0.13 ^Ba^	***
	TBHQV	41.7 ± 0.07 ^Cd^	41.71 ± 0.05 ^Cd^	42.12 ± 0.15 ^Cd^	40.71 ± 0.31 ^Bc^	38.97 ± 0.32 ^Cb^	38.31 ± 0.24 ^Ca^	***
	control 1	40.62 ± 0.37 ^Ab^	41.38 ± 0.02 ^BCb^	40.95 ± 0.82 ^Bb^	40.87 ± 0.23 ^Bb^	37.80 ± 0.01 ^Aa^	37.70 ± 0.08 ^Ba^	***
	control 2	40.29 ± 0.01 ^Ad^	40.88 ± 0.36 ^Abe^	39.45 ± 0.13 ^Ac^	39.62 ± 0.33 ^Ac^	38.32 ± 0.27 ^Bb^	37.43 ± 0.07 ^Aa^	***
	Sign.	***	**	***	***	***	***	
Linoleic acid (C18:2)	BHTV	14.88 ± 0.04 ^Cd^	14.30 ± 0.04 ^Aa^	14.48 ± 0.04 ^Bb^	14.73 ± 0.07 ^Cc^	15.51 ± 0.13 ^Abe^	15.79 ± 0.07 ^Bf^	***
	ETV	14.45 ± 0.04 ^Bb^	15.22 ± 0.12 ^Dc^	13.97 ± 0.05 ^Aa^	14.54 ± 0.05 ^Bb^	15.50 ± 0.18 ^ABd^	15.53 ± 0.07 ^Ad^	***
	PEPV	15.50 ± 0.03 ^Ec^	15.12 ± 0.04 ^CDb^	14.87 ± 0.06 ^Ca^	15.40 ± 0.02 ^Ec^	16.25 ± 0.16 ^Cd^	15.42 ± 0.10 ^Ac^	***
	TBHQV	14.28 ± 0.01 ^Ac^	14.30 ± 0.05 ^Ac^	13.95 ± 0.06 ^Aa^	14.11 ± 0.14 ^Aab^	15.38 ± 0.14 ^Ad^	15.38 ± 0.09 ^Ad^	***
	control 1	14.92 ± 0.09 ^Cb^	14.70 ± 0.03 ^Bb^	14.96 ± 0.27 ^Cb^	14.01 ± 0.05 ^Aa^	15.56 ± 0.05 ^ABc^	15.36 ± 0.06 ^Ac^	***
	control 2	15.39 ± 0.01 ^Dbc^	15.04 ± 0.09 ^Ca^	15.55 ± 0.11 ^Dc^	15.20 ± 0.13 ^Dab^	15.79 ± 0.19 ^Bd^	16.50 ± 0.08 ^Ce^	***
	Sign.	***	***	***	***	***	***	
ΣUFA ^4^	BHTV	55.35 ± 0.04 ^Ac^	55.95 ± 0.06 ^d^	54.94 ± 0.05 ^b^	54.82 ± 0.17 ^b^	52.97 ± 0.20 ^Aa^	53.17 ± 0.23 ^Aa^	***
	ETV	55.53 ± 0.01 ^ABd^	55.68 ± 0.36 ^d^	55.63 ± 0.08 ^d^	55.09 ± 0.22 ^c^	53.75 ± 0.18 ^BCb^	53.36 ± 0.03 ^Aa^	***
	PEPV	55.86 ± 0.09 ^Bd^	56.09 ± 0.34 ^d^	55.34 ± 0.15 ^c^	55.40 ± 0.22 ^c^	54.13 ± 0.09 ^Cb^	53.24 ± 0.08 ^Aa^	***
	TBHQV	55.98 ± 0.08 ^Bc^	56.01 ± 0.04 ^c^	56.08 ± 0.21 ^c^	54.82 ± 0.44 ^b^	54.34 ± 0.45 ^Cb^	53.68 ± 0.33 ^Ba^	***
	control 1	55.54 ± 0.45 ^ABbc^	56.31 ± 0.02 ^c^	55.90 ± 1.09 ^bc^	54.89 ± 0.27 ^b^	53.37 ± 0.05 ^Aba^	53.06 ± 0.06 ^Aa^	***
	control 2	55.68 ± 0.01 ^ABc^	55.91 ± 0.28 ^c^	55.00 ± 0.24 ^b^	54.82 ± 0.46 ^b^	54.11 ± 0.46 ^Ca^	53.94 ± 0.08 ^Ba^	***
	Sign.	*	n.s.	n.s.	n.s.	**	***	
ΣSFA ^5^	BHTV	44.65 ± 0.04 ^Bb^	44.05 ± 0.06 ^a^	45.06 ± 0.05 ^c^	45.18 ± 0.17 ^c^	47.03 ± 0.20 ^Bd^	46.83 ± 0.23 ^Bd^	***
	ETV	44.47 ± 0.01 ^Aba^	44.32 ± 0.36 ^a^	44.37 ± 0.08 ^a^	44.91 ± 0.22 ^b^	46.25 ± 0.18 ^ABc^	46.64 ± 0.03 ^Bd^	***
	PEPV	44.14 ± 0.09 ^Aa^	43.91 ± 0.34 ^a^	44.66 ± 0.15 ^b^	44.60 ± 0.22 ^b^	45.87 ± 0.09 ^Ac^	46.76 ± 0.08 ^Bd^	***
	TBHQV	44.02 ± 0.08 ^Aa^	43.99 ± 0.04 ^a^	43.92 ± 0.21 ^a^	45.18 ± 0.44 ^b^	45.66 ± 0.45 ^Ab^	46.32 ± 0.33 ^Ac^	***
	control 1	44.46 ± 0.45 ^Aab^	43.69 ± 0.02 ^a^	44.10 ± 1.09 ^ab^	45.11 ± 0.27 ^b^	46.63 ± 0.05 ^BCc^	46.94 ± 0.06 ^Bc^	***
	control 2	44.32 ± 0.01 ^Aba^	44.09 ± 0.28 ^a^	45.00 ± 0.24 ^b^	45.18 ± 0.46 ^b^	45.89 ± 0.46 ^Ac^	46.06 ± 0.08 ^Ac^	***
	Sign.	*	n.s.	n.s.	n.s.	**	***	
UFA/SFA	BHTV	1.24 ± 0.00 ^Ac^	1.27 ± 0.00 ^d^	1.22 ± 0.00 ^b^	1.21 ± 0.01 ^b^	1.13 ± 0.01 ^Aa^	1.14 ± 0.01 ^Aa^	***
	ETV	1.25 ± 0.00 ^ABd^	1.26 ± 0.02 ^d^	1.25 ± 0.00 ^d^	1.23 ± 0.01 ^c^	1.16 ± 0.01 ^BCb^	1.14 ± 0.00 ^Aa^	***
	PEPV	1.27 ± 0.00 ^Bd^	1.28 ± 0.02 ^d^	1.24 ± 0.01 ^c^	1.24 ± 0.01 ^c^	1.18 ± 0.00 ^Cb^	1.14 ± 0.00 ^Aa^	***
	TBHQV	1.27 ± 0.00 ^Bc^	1.27 ± 0.00 ^c^	1.28 ± 0.01 ^c^	1.21 ± 0.02 ^b^	1.19 ± 0.02 ^Cb^	1.16 ± 0.02 ^Ba^	***
	control 1	1.25 ± 0.02 ^ABbc^	1.29 ± 0.00 ^c^	1.27 ± 0.06 ^bc^	1.22 ± 0.01 ^b^	1.14 ± 0.00 ^Aba^	1.13 ± 0.00 ^Aa^	***
	control 2	1.26 ± 0.00 ^ABc^	1.27 ± 0.01 ^c^	1.22 ± 0.01 ^b^	1.21 ± 0.02 ^b^	1.18 ± 0.02 ^Ca^	1.17 ± 0.00 ^Ba^	***
	Sign.	*	n.s.	n.s.	n.s.	***	**	

^1^ Different capital superscripts (^ABCDE^) after the mean in the same column indicate significant differences *p* < 0.05, different lowercase letter superscripts (^abcdef^) after the mean in the same row indicate significant differences *p* < 0.05. ^2^ BHTV = 0.2% BHT + vacuum-filling nitrogen packaging; ETV = 0.5% ET + vacuum-filling nitrogen packaging; PEPV = 0.5% PEP + vacuum-filling nitrogen packaging; TBHQV = 0.2% TBHQ + vacuum-filling nitrogen packaging; control 1 = no antioxidants + vacuum-filling nitrogen packaging; control 2 = no antioxidant + non vacuum sealed packaging. ^3^ Sign.: Significance; n.s.: Not significant; *, ** and *** indicate significance at *p* < 0.05, *p* < 0.01 and *p* < 0.001, respectively. ^4^ “UFA” indicates unsaturated fatty acids. ^5^ “SFA” indicates saturated fatty acids.

**Table 3:** The contents of free fatty acids (FFAs) in deep fired *Gryllus bimaculatus* during storage (mg/100 g) ^1^.

Free Fatty Acid	Treatments ^2^	Storage Time/Day						Sign. ^3^
		0	30	60	90	120	150	
Palmitic acid (C16:0)	BHTV	39.91 ± 2.66 ^Ba^	46.98 ± 1.82 ^Cab^	52.76 ± 5.47 ^ABbc^	53.86 ± 1.15 ^Bbc^	59.32 ± 7.50 ^ABc^	55.43 ± 0.94 ^ABbc^	**
	ETV	29.64 ± 1.00 ^Aa^	42.22 ± 0.35 ^ABb^	56.56 ± 5.71 ^ABc^	58.07 ± 0.48 ^Bc^	57.92 ± 3.48 ^ABc^	63.90 ± 5.96 ^Bc^	***
	PEPV	30.75 ± 3.31 ^Aa^	44.10 ± 5.51 ^ABb^	46.76 ± 1.00 ^Ab^	61.92 ± 5.76 ^Bc^	52.82 ± 6.57 ^Abc^	62.91 ± 3.46 ^Bc^	***
	TBHQV	33.15 ± 0.72 ^Aa^	36.09 ± 1.46 ^Aa^	46.38 ± 6.37 ^Ab^	44.73 ± 5.96 ^Ab^	53.83 ± 4.13 ^Ab^	47.91 ± 4.28 ^Ab^	**
	control 1	50.23 ± 3.82 ^Ca^	47.05 ± 3.62 ^Ca^	63.49 ± 6.62 ^BCbc^	59.01 ± 0.1 ^Bb^	68.72 ± 4.66 ^BCc^	79.63 ± 3.88 ^Cd^	***
	control 2	54.45 ± 1.39 ^Ca^	64.25 ± 6.69 ^Dab^	72.57 ± 3.42 ^Cbc^	62.52 ± 5.37 ^Bab^	76.41 ± 5.18 ^Cc^	92.68 ± 6.58 ^Dd^	***
	Sign. ^2^	***	***	***	**	**	***	
Stearic acid (C18:0)	BHTV	14.83 ± 0.67 ^Aa^	20.86 ± 2.08 ^BCb^	27.27 ± 4.54 ^Cc^	27.74 ± 0.69 ^ABc^	19.52 ± 1.37 ^ABb^	25.27 ± 1.96 ^Bc^	**
	ETV	14.23 ± 0.66 ^Aa^	17.61 ± 0.42 ^ABa^	13.35 ± 1.55 ^Aa^	23.78 ± 1.56 ^Ab^	17.61 ± 3.41 ^Aa^	37.82 ± 4.51 ^Cc^	***
	PEPV	13.55 ± 4.20 ^Aa^	17.47 ± 1.50 ^ABa^	23.94 ± 0.47 ^BCb^	29.05 ± 3.39 ^Bb^	16.93 ± 1.75 ^Cb^	15.20 ± 1.19 ^Aa^	***
	TBHQV	16.73 ± 0.65 ^ABb^	11.74 ± 1.70 ^Aa^	19.88 ± 3.29 ^Bb^	23.38 ± 1.48 ^Ab^	23.12 ± 2.14 ^BCb^	21.82 ± 4.43 ^ABb^	**
	control 1	20.07 ± 1.76 ^Ba^	21.76 ± 1.22 ^BCa^	19.21 ± 0.87 ^Ba^	35.82 ± 0.23 ^Cc^	25.87 ± 1.13 ^Cb^	19.12 ± 1.86 ^ABa^	***
	control 2	24.61 ± 0.57 ^Ca^	26.93 ± 6.48 ^Ca^	28.30 ± 1.95 ^Ca^	37.44 ± 3.54 ^Cb^	31.56 ± 1.55 ^Dab^	47.15 ± 3.60 ^Dc^	***
	Sign.	***	**	***	***	***	***	
Oleic acid (C18:1)	BHTV	16.36 ± 2.33 ^Aa^	23.64 ± 5.97 ^ABab^	18.61 ± 0.70 ^Aa^	27.53 ± 0.46 ^ABb^	27.74 ± 4.63 ^Ab^	24.58 ± 3.08 ^Aab^	**
	ETV	14.86 ± 0.74 ^Aa^	16.89 ± 0.11 ^Aa^	35.83 ± 1.29 ^BCb^	18.69 ± 4.12 ^Aa^	30.11 ± 6.65 ^Ab^	31.04 ± 4.98 ^ABb^	***
	PEPV	14.71 ± 4.55 ^Aa^	22.76 ± 7.40 ^ABab^	27.75 ± 0.99 ^Bab^	25.09 ± 7.66 ^ABab^	25.48 ± 5.43 ^Aab^	33.75 ± 3.89 ^ABb^	*
	TBHQV	15.88 ± 0.51 ^Aa^	15.35 ± 4.56 ^Aa^	16.52 ± 2.24 ^Aa^	21.72 ± 3.68 ^ABa^	20.36 ± 2.48 ^Aa^	47.38 ± 13.54 ^Bb^	***
	control 1	20.54 ± 2.32 ^Aa^	23.38 ± 2.95 ^ABab^	28.26 ± 4.71 ^Bbc^	32.72 ± 0.18 ^Cc^	28.68 ± 2.06 ^Abc^	39.45 ± 4.68 ^ABd^	***
	control 2	24.61 ± 0.57 ^Ca^	26.93 ± 6.48 ^Ca^	28.30 ± 1.95 ^Ca^	37.44 ± 3.54 ^Cb^	31.56 ± 1.55 ^Dab^	47.15 ± 3.60 ^Dc^	***
	Sign.	***	*	***	**	***	*	
Linoleic acid (C18:2)	BHTV	11.39 ± 0.86 ^Ba^	22.25 ± 3.19 ^Bc^	16.55 ± 1.17 ^ABb^	25.1 ± 0.32 ^Bc^	29.79 ± 2.93 ^Bd^	24.81 ± 2.17 ^Bc^	***
	ETV	11.49 ± 0.64 ^Ba^	13.64 ± 0.15 ^ABa^	11.23 ± 2.99 ^Aa^	16.34 ± 3.97 ^Aa^	27.57 ± 4.08 ^Bb^	35.08 ± 1.88 ^Cc^	***
	PEPV	8.05 ± 0.20 ^Aa^	13.61 ± 2.39 ^ABb^	15.48 ± 0.14 ^ABb^	16.42 ± 2.97 ^Ab^	24.31 ± 1.90 ^Bc^	28.23 ± 3.52 ^BCc^	***
	TBHQV	11.19 ± 0.15 ^Bb^	6.12 ± 0.30 ^Aa^	12.83 ± 1.40 ^Abc^	16.44 ± 1.52 ^Abc^	14.10 ± 4.54 ^Abc^	17.77 ± 3.29 ^Ac^	**
	control 1	17.01 ± 1.03 ^Ca^	20.52 ± 3.20 ^Ba^	18.96 ± 1.78 ^Ba^	18.32 ± 0.07 ^Aa^	25.73 ± 7.61 ^Bab^	29.02 ± 4.12 ^BCb^	*
	control 2	41.48 ± 0.90 ^Dab^	48.32 ± 8.70 ^Cab^	39.79 ± 3.80 ^Ca^	53.57 ± 3.62 ^Cb^	45.80 ± 1.38 ^Cab^	49.83 ± 5.18 ^Dab^	*
	Sign.	***	***	***	***	***	***	
ΣUFA ^4^	BHTV	27.74 ± 2.82 ^Aa^	45.89 ± 9.11 ^Bb^	35.16 ± 1.85 ^ABa^	52.64 ± 0.19 ^Bb^	57.54 ± 7.56 ^Bb^	49.40 ± 5.17 ^Ab^	***
	ETV	26.36 ± 1.36 ^A^	30.53 ± 0.21 ^ABa^	47.06 ± 1.79 ^Cab^	35.03 ± 8.08 ^Aa^	57.68 ± 6.83 ^Bc^	66.12 ± 6.84 ^Ac^	***
	PEPV	22.76 ± 4.71 ^Aa^	36.36 ± 8.04 ^ABb^	43.24 ± 0.91 ^BCb^	41.51 ± 10.55 ^ABb^	49.79 ± 7.32 ^ABbc^	61.98 ± 7.26 ^Ac^	**
	TBHQV	27.07 ± 0.59 ^Aa^	21.46 ± 4.35 ^Aa^	29.35 ± 3.58 ^Aa^	38.15 ± 5.20 ^ABa^	34.46 ± 6.00 ^Aa^	65.16 ± 14.53 ^Ab^	***
	control 1	37.56 ± 3.32 ^Ba^	43.90 ± 4.96 ^Bab^	47.22 ± 6.48 ^Cab^	51.04 ± 0.16 ^Bab^	54.41 ± 9.20 ^Bb^	68.46 ± 8.79 ^Ac^	**
	control 2	73.24 ± 1.41 ^Ca^	79.55 ± 11.58 ^Ca^	78.77 ± 8.47 ^Da^	85.88 ± 6.03 ^Ca^	110.47 ± 13.87 ^Cb^	87.69 ± 8.79 ^Ba^	**
	Sign.	***	***	***	***	***	**	
ΣSFA ^5^	BHTV	54.73 ± 2.34 ^Ba^	67.84 ± 3.63 ^Bb^	80.04 ± 9.90 ^Ab^	81.6 ± 1.44 ^Bb^	78.84 ± 8.85 ^Ab^	80.70 ± 2.90 ^Ab^	**
	ETV	43.87 ± 1.56 ^Aa^	59.83 ± 0.75 ^ABb^	69.92 ± 7.17 ^A^	81.84 ± 1.69 ^Bd^	75.54 ± 0.39 ^Acd^	101.72 ± 10.45 ^Be^	***
	PEPV	44.29 ± 5.23 ^Aa^	61.57 ± 6.55 ^ABb^	70.70 ± 1.44 ^Abc^	90.97 ± 4.94 ^BCd^	79.75 ± 8.32 ^Ac^	78.10 ± 4.44 ^Ac^	***
	TBHQV	19.89 ± 0.85 ^ABa^	47.83 ± 2.87 ^Aa^	66.26 ± 9.64 ^Ab^	68.11 ± 7.15 ^Ab^	76.95 ± 5.86 ^Ab^	69.73 ± 6.79 ^Ab^	***
	control 1	70.30 ± 5.15 ^Ca^	68.82 ± 4.84 ^Ba^	82.70 ± 7.49 ^Ab^	94.83 ± 0.14 ^Cc^	94.59 ± 5.48 ^Bc^	98.76 ± 5.63 ^Bc^	***
	control 2	79.07 ± 1.71 ^Da^	91.19 ± 13.10 ^Cab^	100.87 ± 4.46 ^Bb^	99.96 ± 8.90 ^Cb^	107.98 ± 5.50 ^Cb^	139.84 ± 10.17 ^Cc^	***
	Sign.	***	***	***	***	***	***	
Total FFAs	BHTV	82.48 ± 5.16 ^Ba^	113.73 ± 12.01 ^Bb^	115.20 ± 11.20 ^ABb^	134.24 ± 1.52 ^BCb^	136.37 ± 16.18 ^ABb^	130.10 ± 7.84 ^Ab^	***
	ETV	70.23 ± 2.91 ^ABa^	90.36 ± 0.96 ^ABb^	116.98 ± 8.88 ^ABc^	116.87 ± 9.57 ^ABc^	133.22 ± 7.23 ^ABc^	167.84 ± 16.30 ^Ad^	***
	PEPV	67.06 ± 9.82 ^Aa^	97.93 ± 14.30 ^ABb^	113.94 ± 2.35 ^ABbc^	132.48 ± 9.66 ^BCc^	129.54 ± 15.61 ^ABc^	140.08 ± 10.33 ^Ac^	***
	TBHQV	76.95 ± 0.72 ^ABab^	69.29 ± 6.85 ^Aa^	95.61 ± 11.58 ^Abc^	106.26 ± 11.24 ^Ac^	111.41 ± 9.00 ^Ac^	134.89 ± 20.02 ^Ad^	***
	control 1	107.86 ± 7.97 ^Ca^	112.72 ± 9.78 ^Ba^	129.92 ± 13.98 ^Bab^	145.87 ± 0.26 ^Cbc^	149.00 ± 14.61 ^Bbc^	167.22 ± 13.46 ^Ac^	***
	control 2	152.30 ± 3.11 ^Da^	170.73 ± 24.56 ^Ca^	179.63 ± 12.49 ^Ca^	185.84 ± 14.85 ^Da^	218.45 ± 17.46 ^Cb^	227.53 ± 18.91 ^Bb^	**
	Sign.	***	***	***	***	***	***	

^1^ Different capital superscripts (^ABCDE^)after the mean in the same column indicate significant differences *p* < 0.05, different small letter superscripts (^abcdef^) after the mean in the same row indicate significant differences *p* < 0.05; ^2^ BHTV = 0.2% BHT + vacuum-filling nitrogen packaging; ETV = 0.5% ET + vacuum-filling nitrogen packaging; PEPV = 0.5% PEP + vacuum-filling nitrogen packaging; TBHQV = 0.2% TBHQ + vacuum-filling nitrogen packaging; control 1= no antioxidants + vacuum-filling nitrogen packaging; control 2 = no antioxidant + non vacuum sealed packaging; ^3^ Sign.: Significance; n.s.: not Significant; *, ** and *** indicate significance at *p* < 0.05, *p* < 0.01 and *p* < 0.001, respectively. ^4^ “UFA” indicates unsaturated fatty acids. ^5^ “SFA” indicates saturated fatty acids.

## Data Availability

All data and materials are available on request.
